# Silicea

**Published:** 1898-05

**Authors:** 


### HOMŒOPATHIC PHYSICIAN,

A MONTHLY JOURNAL OF

HOMŒOPATHIC MATERIA MEDICA AND CLINICAL MEDICINE.

"If our school ever gives up the strict inductive method of Hahnemann, we are lost, and deserve only to be mentioned as a caricature in the history of medicine."—CONSTANTINE HERING.

Vol. XVIII. MAY, 1898. No. 5.

### EDITORIAL.

SILICEA.—Sweat of the feet offensive, causing soreness between the toes. There is no remedy so frequently indicated in this condition as Silicea. The editor has frequently verified it in his own practice. Dr. Lippe gave several other remedies as being useful also in this condition; they are, Kali-carb. Graphites, Lycopodium, Nitric-acid. There are, however, many other remedies that have the condition. A Repertory of foot sweat, by Dr. Olin M. Drake was published in THE HOMŒOPATHIC PHYSICIAN for June, 1894. This gives all that is known about foot sweat according to the materia medica, up to date. Reprints of this Repertory may be had at this office. Before leaving this subject we ought to mention Dr. Guernsey's keynote for Calcarea-carbonica, "Cold feet as if the patient had on cold damp stockings."

Silicea has sense of constriction of external parts.

Silicea is a useful remedy in epileptic attacks, especially when they occur at night during the new moon. Kali-carb. and Calcarea-carb. are useful remedies, according to Dr.

Lippe, in epilepsy. Bufo is, however, the principal remedy in this terrible disease.

The editor of this journal has had much success in a case of epilepsy with Nitric-acid.

The patient was a young girl who had the epileptic attacks every month at the access of the menstrual flow, although they were not wanting in the intervals between the menstrual periods. At each attack the patient would fall down with a scream, and then go through the usual phenomena of the convulsions, during which both arms would become dislocated at the shoulder-joints. The patient would come out of these seizures with the exclamation, "What did you say?" and then a physician had to be called to reduce the luxation. The patient had probably been treated with Mercury before the editor had charge of her case. Her tongue was deeply indented upon the edges, and with sore pimples upon the tip. Every day she had numerous insensible spells, called by pathologists "*petit mal.*" These insensible spells would amount to as many as fifty in one day. Six years ago she came under the care of the writer, and he gave her a number of remedies without any perceptible effect until, after prolonged study of the case, he decided upon Nitric-acid. This was given in the two hundredth potency in occasional doses and persisted in, up to the present time. Then the spasms were found to come at longer intervals. Instead of once a month, they came once in two months, then once in three months. Later they came once in five months, then once in six months, then once a year. At the time of writing this editorial there has been no spasm for one year and three months, and the spells of *petit mal* have been reduced to two or three a day, while some days pass without any at all! The bowels, which were obstinately blocked, are now regular and the little woman is bright and hopeful and making herself exceedingly useful among her friends with whom she resides. It is to be observed that conditions sometimes arose which would necessitate the giving of other remedies which might be indicated. But these occasions were rare, and as a rule she took nothing but Nitric-acid for the whole period she has been under our treatment.

Another observation may be made. In the last two or three spasms which she suffered there was no dislocation of the two humeri.

The Silicea patient takes cold from having the head uncovered. Belladonna has cold in the head from having the hair cut.

Silicea patient takes cold from any exposure of the feet, such as getting the feet wet.

Silicea has ebullitions and thirst from drinking small quantities of wine.

Zinc has headache from small quantities of wine. For a full list of indications for effects from wine see the editorial on Conium in THE HOMŒOPATHIC PHYSICIAN for January, 1897, pages 2 and 3.

Silicea is indicated in children who are slow in learning to walk. Calcarea-carb. is similarly indicated.

The Silicea patient is sleepy but is unable to sleep. This is similar to Belladonna. One of the prime indications of Belladonna is drowsiness with all ailments. Calcarea-carb. has sleep prevented by many thoughts coming into the mind.

Nux-moschata has great sleepiness with deep sleep at all hours of the day. The patient falls asleep the moment he becomes quiet.

The Silicea patient has many dreams. So has Aconite. There are many other remedies which are indicated for dreaming, but Dr. Lippe did not give the indications. Those who are desirous of full information on this point may consult Dr. Knerr's excellent Repertory to *The Guiding Symptoms*, page 1050.

